# Detection of methicillin-resistant *Staphylococcus aureus* using phage amplification combined with matrix-assisted laser desorption/ionization mass spectrometry

**DOI:** 10.1007/s00216-016-0070-3

**Published:** 2016-11-19

**Authors:** Jon C. Rees, John R. Barr

**Affiliations:** Division of Laboratory Sciences, National Center for Environmental Health, Centers for Disease Control and Prevention, Chamblee, GA 30341 USA

**Keywords:** MRSA, Phage amplification, Antibiotic resistance, MALDI, Biotyper, *Staphylococcus aureus*

## Abstract

Antibiotic resistance continues to contribute significantly to morbidity and mortality across the world. Developing new tests for antibiotic-resistant bacteria is a core action to combat resistant infections. We describe a method that uses phage amplification detection (PAD) combined with matrix-assisted laser desorption/ionization mass spectrometry (MALDI-MS) to rapidly identify *Staphylococcus aureus* and determine phenotypic susceptibility to cefoxitin. Samples tested for *S. aureus* are incubated together with bacteriophage in the presence and absence of cefoxitin and subjected to rapid trypsin digestion followed by MALDI-MS analysis. Tryptic peptides derived from amplified phage proteins can be detected by MALDI-MS, as validated by time-of-flight (TOF)/TOF analysis of each peptide combined with database searching. Methicillin-resistant *S. aureus* show significant phage amplification in the presence of cefoxitin, while methicillin-sensitive *S. aureus* show no phage amplification relative to a no-antibiotic control. We also show that PAD methodology can be implemented on an FDA-approved commercial MALDI-MS bacterial identification system to identify *S. aureus* and determine antibiotic susceptibility. The novelty of this assay includes the use of phage-derived tryptic peptides as detected by MALDI-MS to monitor the results of PAD on an instrument common to many modern microbiology laboratories.

**Electronic supplementary material:**

The online version of this article (doi:10.1007/s00216-016-0070-3) contains supplementary material, which is available to authorized users.

## Introduction

In 2013, the Centers for Disease Control and Prevention issued a detailed report on the specter of antibiotic resistance threats in the USA, describing the substantial burden of current and emerging antibiotic-resistant strains of bacteria imposed on the US healthcare system [1]. Annually, in the USA, at least two million people contract infections from antibiotic-resistant bacteria, resulting in approximately 23,000 deaths. Direct healthcare costs in the USA related to antibiotic-resistant bacteria are as high as $20 billion, with estimates for lost productivity ranging up to $35 billion [1]. While the report focuses on antibiotic threats within the USA, it also recognizes the worldwide nature of the antibiotic resistance dilemma. The CDC outlines four core actions to combat antibiotic-resistant infections, which includes “developing new diagnostic tests for resistant bacteria” [1].

Previous studies have demonstrated the feasibility of utilizing phage amplification detection (PAD) combined with mass spectrometry to rapidly detect bacterial species from direct culture [2–6] and determine its antibiotic susceptibility [7]. Bacteriophages are virus particles that can infect a bacterial host, hijack the cell machinery of the host to produce clonal progeny virus particles, and lyse the bacterial host, spilling the newly generated bacteriophages into the surrounding milieu. PAD exploits the specificity of a bacteriophage for its host to initiate a phage infection in the targeted bacterium, followed by monitoring the sample for evidence of a successful bacteriophage infection, which implies the presence of the targeted bacterium. Mass spectrometry functions as a PAD platform by detecting the protein products of new phage replication, which may include both structural and nonstructural protein markers. Antibiotic resistance by PAD is determined by the detection of phage replication in the presence of an antibiotic when compared to a no-antibiotic control.

Matrix-assisted laser desorption/ionization mass spectrometry (MALDI-MS) has been developed and introduced as a commercial tool for the rapid detection of bacteria [8–10], with efforts being made to expand its capabilities to include antibiotic resistance determination [11–14]. MALDI-MS is an attractive platform for bacterial detection because of the relative ease of sample preparation in combination with its ability to analyze large numbers of samples in a short period of time. While PAD combined with liquid chromatography-mass spectrometry (LC-MS) techniques can quantitatively identify bacteria and determine antibiotic susceptibility, the serial nature of running samples on a liquid chromatography system—however short the LC run—may limit the routine use of PAD-LC-MS in a clinical setting. Furthermore, two MALDI time-of-flight (TOF)-MS systems have been approved by the FDA for routine identification of bacteria and are rapidly being deployed in clinical laboratories. Thus, clinically relevant MALDI-MS assay development is of growing importance.

This study demonstrates PAD as a tool to determine the presence of *Staphylococcus aureus* in a sample and determine its antibiotic susceptibility using MALDI-MS as a detector. Using ^15^N-labeled bacteriophage to initiate an infection, we can ascertain the protein products of a successful phage amplification process using MALDI to detect three tryptic peptides of the newly replicated bacteriophage. TOF/TOF analysis of each peptide combined with database searching confirms that the targeted peptides at each mass are derived from the capsid protein of newly generated bacteriophage. PAD of *S. aureus* and antibiotic susceptibility determination by MALDI-MS is shown to work on an FDA-approved platform for bacterial identification.

## Materials and methods

### Bacterial strains and bacteriophage

All bacteria used in this study were purchased from the American Type Culture Collection (ATCC) (Manassas, VA). Antibiotic susceptibility for all strains of bacteria was determined according to the Clinical and Laboratory Standards Institute (CLSI) guidelines using the guiding document M100-S25 as described elsewhere [7]. Details of each strain used and antibiotic susceptibility determination results are shown in Table 1. Bacteriophage K (ATCC-19685-B1) and its host (ATCC-19685) were also purchased from the ATCC and propagated, harvested, and quantified as described elsewhere [7]. ^15^N-labeled bacteriophage K was generated by culturing its host in ^15^N-labeled broth (Cambridge Isotope Laboratories, Inc., Tewksbury, MA) and propagating the phage in this host continually using the ^15^N-labeled broth. Phage samples were stored at 4 °C until needed.

**Table 1:** List of *Staphylococcus aureus* strains used in this study along the antibiotic susceptibly testing results

Strain	Antibiotic susceptibility	MIC to cefoxitin (μg/mL)
ATCC-12598	MSSA	<0.5
ATCC-27694	MSSA	<0.5
BAA-1720	MRSA	>32
BAA-1750	MRSA	>32
ATCC-29213	MSSA	<0.5
ATCC-43300	MRSA	>32

### Phage amplification

Bacterial strains used in PAD experiments were grown overnight in tryptic soy broth. Samples for identification were generated by mixing each bacterium at a final concentration of 2.5 × 10^7^ cfu/mL with ^15^N-labeled bacteriophage at a final concentration of 1 × 10^8^ plaque-forming units (pfu)/mL and incubating at 37 °C for 5 h. Samples for antibiotic susceptibility were generated by mixing bacteria at a final concentration of 2.5 × 10^7^ cfu/mL with cefoxitin at 4 μg/mL and incubating at 37 °C for 2.5 h. Bacteriophage was added to each cefoxitin-containing sample at a final concentration of 1 × 10^8^ pfu/mL and incubated an additional 2.5 h. Controls containing only bacteriophage and only a bacterial strain were incubated along with all samples for 5 h at 37 °C. Following incubation, all samples were centrifuged briefly to remove large debris, filtered through a 30-kDa molecular weight cutoff filter (Amicon Ultra; Millipore, Billerica, MA), and washed twice in the filter with 50 mM ammonium bicarbonate at pH 7.4.

### Trypsin digestion

Ten microliters of each sample was mixed with 10 μL of 0.1% RapiGest (Waters, Inc., Bedford, MA) and heated at 100 °C for 10 min. After the samples cooled, 10 μL of sequence grade trypsin (Promega, Madison, WI) at 0.4 μg/mL was added to each sample and incubated at 52 °C for 3 min for enzymatic digestion. Each sample was processed and purified using a C18 ZipTip (Millipore, Billerica, MA) prior to MALDI plating.

### MALDI-MS analysis

Samples for MALDI-MS analysis using an Applied Biosystems (Framingham, MA) model 4800 MALDI-TOF/TOF instrument were spotted onto a 384-position MALDI plate by mixing 0.5 μL of sample with 0.5 μL of alpha-cyano-4-hydroxycinnamic acid at each spot. Mass spectra of each spot were obtained by scanning 2000 to 3000 *m*/*z* in MS-positive ion reflector mode. Fragment ion spectra were obtained for designated masses using the instrument in TOF/TOF mode. The instrument uses a ND:YAG laser at 355 nm, and each spectrum is the average of 9800 laser shots. Samples for analysis on the Bruker Biotyper were prepared by mixing 1.0 μL of the sample with 1.0 μL of alpha-cyano-4-hydroxycinnamic acid at each spot. The instrument operates in linear mode, and samples were scanned over the range 2000–3000 *m*/*z*.

Peaks from each TOF/TOF fragment ion spectrum were extracted using a Mascot Distiller (Matrix Science, London, UK; version 2.5.1) and searched against a database of bacteriophage K protein sequences culled from the NCBInr database (www.ncbi.nih.gov) using the Mascot search algorithm (Matrix Science, London, UK; version 2.5.1).

## Results and discussion

**Fig. 1:**
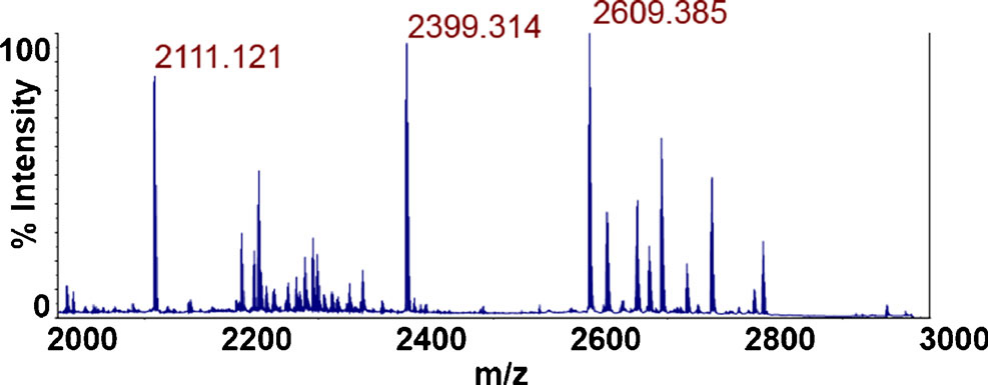
MALDI-MS spectrum of tryptic peptides derived from wild-type bacteriophage K. The peak masses correspond to the following peptide sequences: 2111.121 (SFQTGYGITPDTQIDAGALR), 2399.314 (LSINVNAMYQQQPQFVSIYR), and 2609.385 (GFGTATDAYMPIGVHADFVNSILGR)

**Fig. 2:**
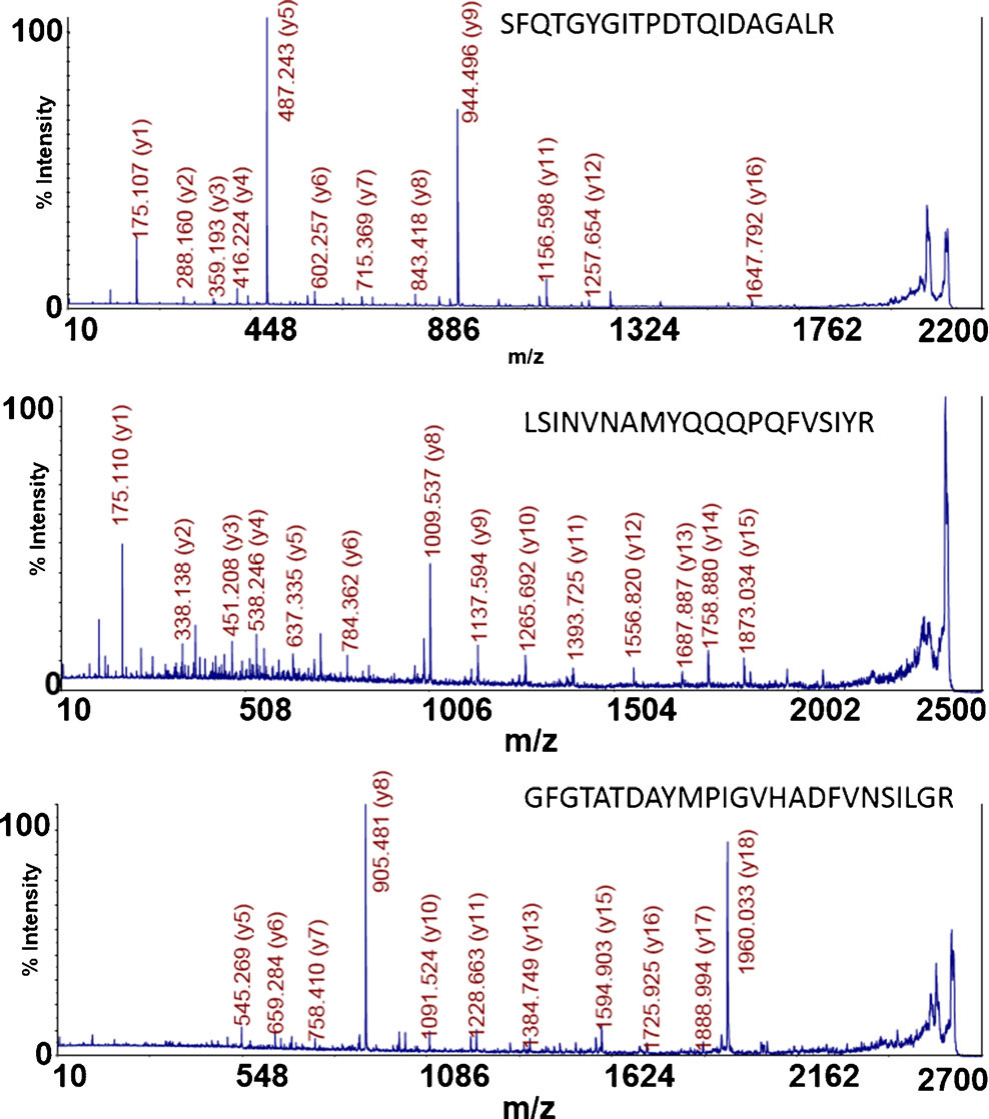
TOF/TOF spectra of three tryptic peptides derived from bacteriophage K

**Fig. 3:**
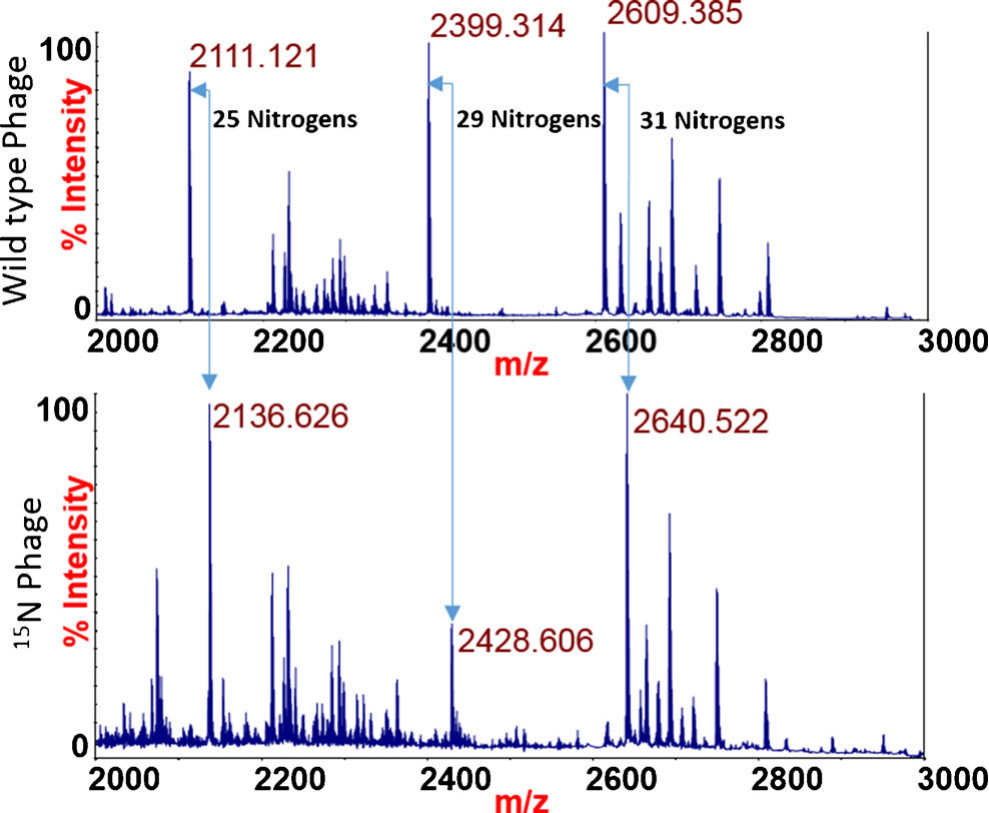
Comparison of wild-type phage K with ^15^N-labeled bacteriophage K. The shift in masses between the wild-type and ^15^N-labeled bacteriophage corresponds precisely to the number of nitrogen atoms in the amino acid sequences of the peptides assigned to each respective mass

The MALDI spectrum of a tryptic digest of isolated wild-type bacteriophage K at 5 × 10^8^ pfu/mL is shown in Fig. 1, with peaks at *m*/*z* that correspond to peptides discovered by LC-MS/MS and database searching highlighted (see Electronic Supplementary Material (ESM) Table S1). Intense peaks at *m*/*z* 2111.1, *m*/*z* 2399.3, and *m*/*z* 2609.4 correspond in mass to the bacteriophage K capsid protein tryptic peptides (K)SFQTGYGITPDTQIDAGALR(R), (K)LSINVNAMYQQQPQFVSIYR(Q), and (K)GFGTATDAYMPIGVHADFVNSILGR(Q), respectively. TOF/TOF analysis of each respective peak is shown in Fig. 2, and database searching of each fragment ion spectrum against a bacteriophage K sequence database resulted in a singular, significant database identification that matched the amino acid sequences of the respective MS peaks. Further evidence that these peaks are tryptic peptides belonging to bacteriophage K is revealed upon comparison of wild-type bacteriophage K with ^15^N-labeled bacteriophage shown in Fig. 3. The mass difference between the peak at *m*/*z* 2111 and *m*/*z* 2136 corresponds with the exact number of nitrogen atoms (25) in the peptide (K)SFQTGYGITPDTQIDAGALR(R). TOF/TOF analysis of the peak at *m*/*z* 2136 reveals a fragment ion spectrum identical to the spectrum derived from the peak at *m*/*z* 2111 after accounting for the number of nitrogen atoms in each fragment. This similar observation can be made for the pairs of peaks at *m*/*z* 2399 and *m*/*z* 2428 and at *m*/*z* 2609 and *m*/*z* 2640.

The detection limit for bacteriophage K was determined by a 50% serial dilution series beginning at 5 × 10^8^ pfu/mL down to 7.8 × 10^6^ pfu/mL. Fig. S1 (see ESM) shows the MALDI spectrum of relevant dilutions, revealing that S/N falls below detection levels at approximately 1 × 10^8^ pfu/mL. Thus, any bacteriophage amplification event must produce at least 1 × 10^8^ pfu/mL of phage K in order to generate enough MALDI-MS signal to be deemed a successful infection and the presence of *S. aureus* established.

**Fig. 4:**
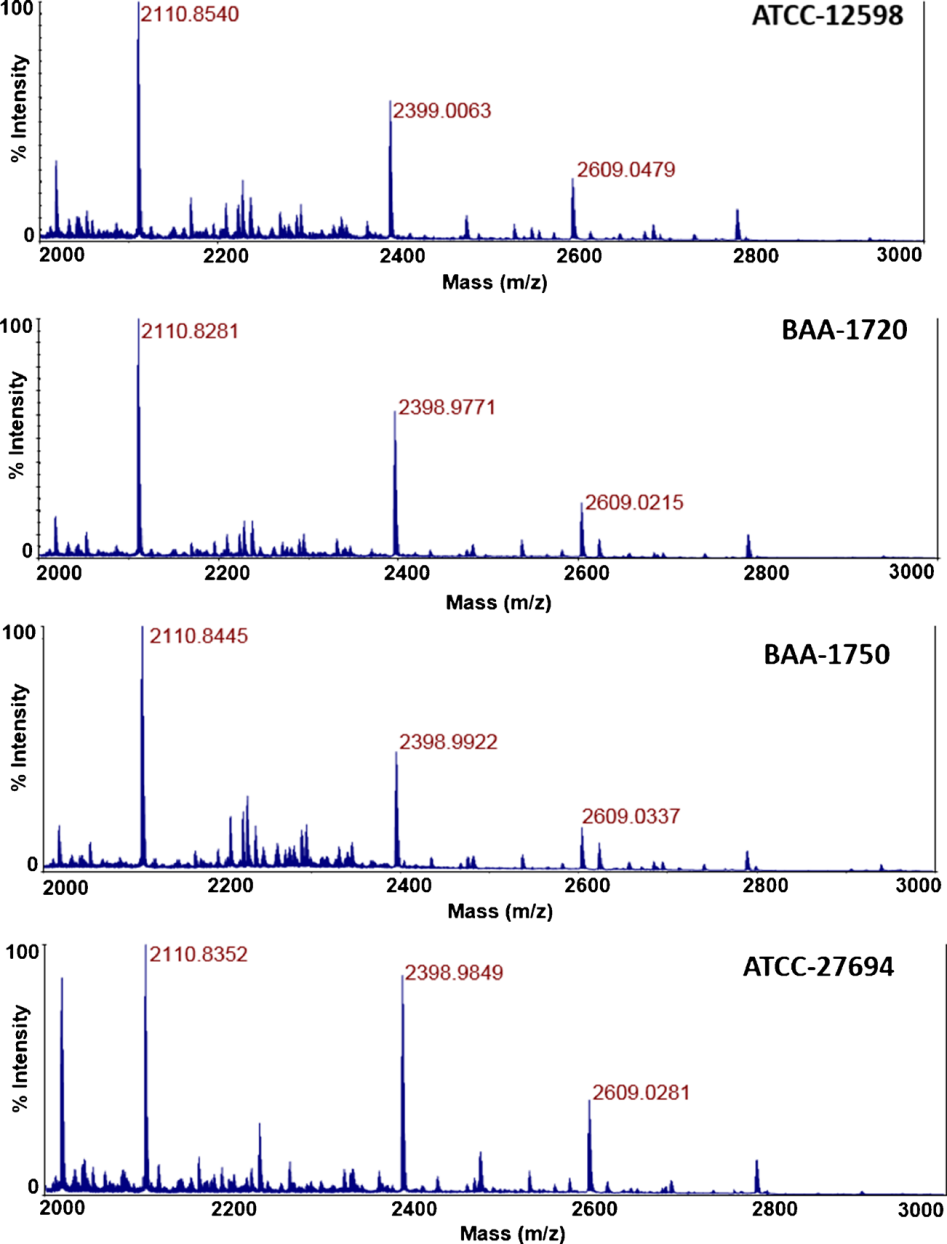
MALDI spectra of four strains of *S. aureus* after a 5-h PAD experiment. Phage amplification is detected in all strains of *S. aureus*

A detection experiment was conducted such that four strains of *S. aureus* (ATCC-12598, ATCC-27694, BAA-1720, and BAA-1750) at 2.5 × 10^7^ cfu/mL were infected with an inoculum of ^15^N bacteriophage at an initial concentration of 1 × 10^8^ pfu/mL. Tryptic peptide peaks associated with newly propagated bacteriophage should contain predominantly ^14^N, because the broth used to culture the bacterial host consists of nutrients with nitrogen isotopes at natural abundances. Figure 4 displays the MALDI spectrum of each respective amplification event, showing the appearance of the established three tryptic bacteriophage peaks at *m*/*z* 2111, *m*/*z* 2399, and *m*/*z* 2609. Mass spectra obtained from controls consisting of only bacteriophage K (no bacteria) and bacteria only (no labeled phage K) carried through the infection and digest procedures revealed no amplified phage peaks and no peaks that may interfere with peaks associated with newly amplified bacteriophage (ESM Fig. S2). Thus, the presence of *S. aureus* in the respective cultures can be established after a 5-h infection process based upon the detection of phage amplification.

**Fig. 5:**
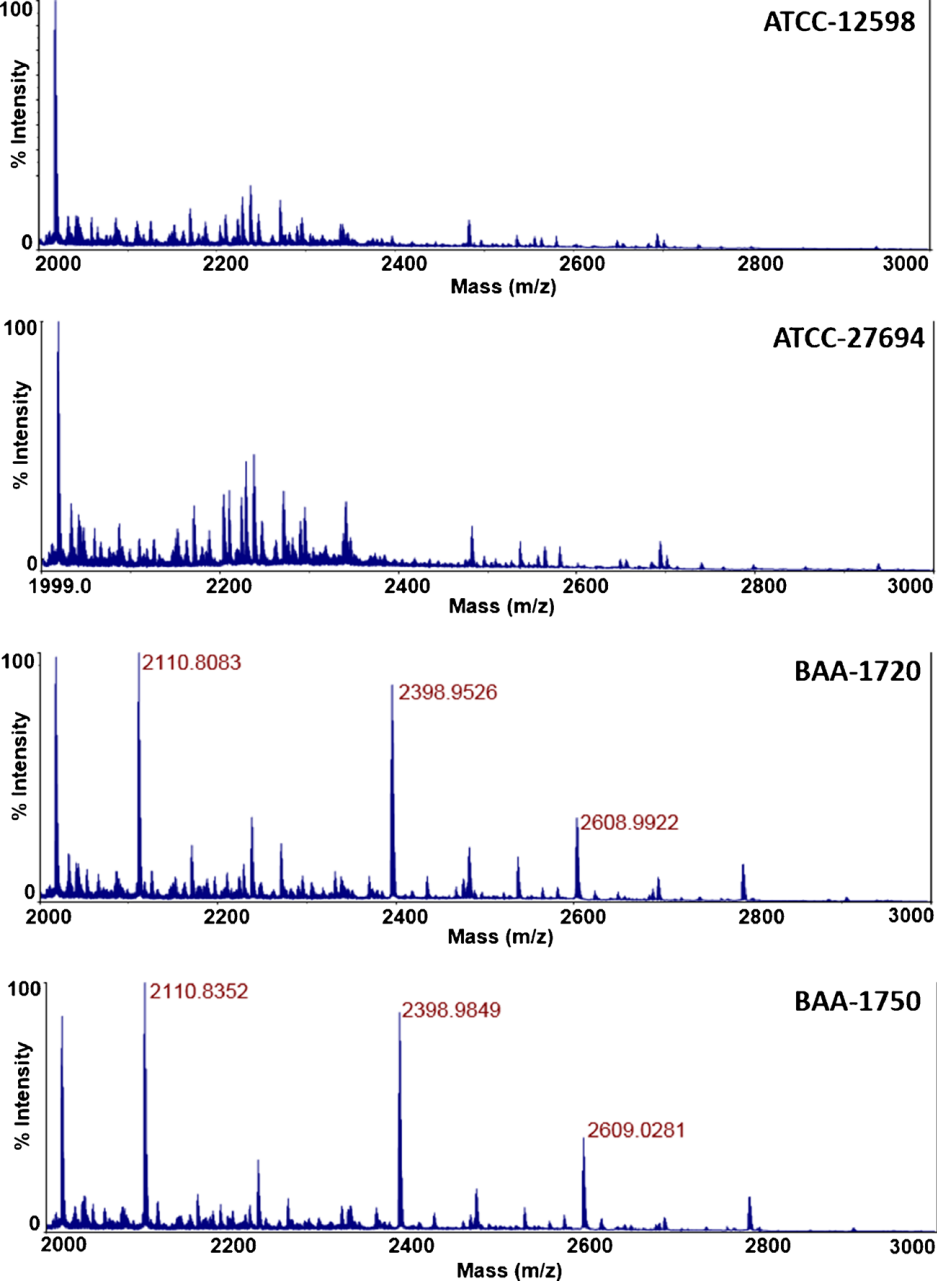
MALDI spectra of four strains of *S. aureus* after a 5-h PAD amplification experiment in the presence of 4 μg/mL cefoxitin. Phage amplification can be seen in methicillin-resistant bacteria BAA-1720 and BAA-1750, while no amplification is noted in the methicillin-sensitive *S. aureus* strains

Antibiotic susceptibility of the four strains of *S. aureus* to 4 μg/mL cefoxitin was determined by carrying out a bacteriophage amplification experiment in the presence of the antibiotic with comparison to a no-antibiotic phage amplification infection as demonstrated above. Because bacteriophage can replicate only in living bacteria, no phage amplification—and therefore no phage proteins—is expected in antibiotic susceptible *S. aureus* strains. Figure 5 shows the side-by-side comparison of each respective *S. aureus* strain after phage amplification with the no-antibiotic sample and in the presence of 4 μg/mL cefoxitin. For the two methicillin-sensitive *S. aureus* (MSSA) strains—ATCC-12598 and ATCC 27694—significant amplification can be seen in the no-antibiotic samples as shown in Fig. 4, while no phage-based tryptic peaks can be detected in the sample with cefoxitin; phage replication did not occur to produce the ^14^N-labeled progeny phage, and the ^15^N parent phage is below the limit of detection of the MALDI-TOF-MS. Therefore, not only can the presence of *S. aureus* can be determined in these samples, but they are also susceptible to cefoxitin. The methicillin-resistant *S. aureus* (MRSA) strains BAA-1720 and BAA-1750 show significant phage amplification in both the no-antibiotic and the cefoxitin-containing samples, indicating that live *S. aureus* bacteria in the cefoxitin-containing sample were responsible for the phage amplification. Thus, the presence of *S. aureus* is established in these two samples, and their susceptibility to cefoxitin is confirmed.

**Fig. 6:**
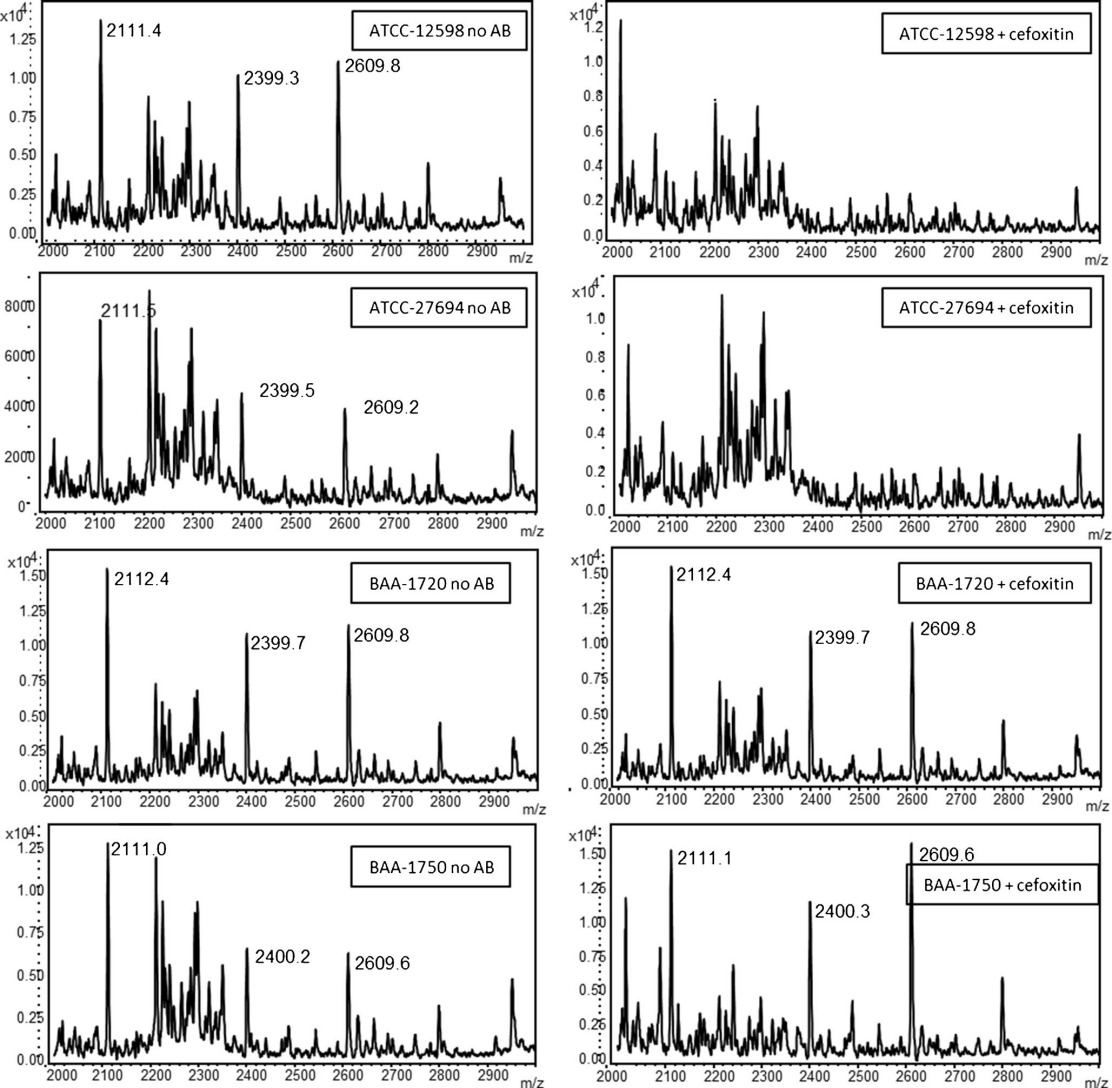
MALDI-MS spectra of PAD with four strains of *S. aureus* with and without antibiotic (4 μg/mL cefoxitin) obtained on a Bruker Biotyper instrument

While the above experimentation for PAD combined with MALDI was conducted on a research grade instrument with TOF/TOF capabilities in reflector mode, MALDI instrumentation in clinical laboratories often consists of instruments that function in linear ion mode without a reflectron. Because of the expanding use and distribution of these commercial instruments, it would be useful to establish the use of PAD as detected on contemporary instrumentation such as the Bruker Biotyper or the BioMerieux VITEK MS that have been approved by the FDA for routine bacterial identification in clinical laboratories. Figure 6 shows the MALDI-MS spectra obtained on a Bruker Biotyper of the phage amplification tryptic products of the four *S. aureus* strains used in this study incubated in the no-antibiotic sample and in the presence of 4 μg/mL cefoxitin. While the MSSA strains ATCC-12598 and ATCC-27694 show clear phage amplification as determined by the presence of the peaks at *m*/*z* 2111, *m*/*z* 2399, and *m*/*z* 2609 in the sample with no antibiotic, the spectra of the phage amplification products in the presence of 4 μg/mL cefoxitin show no peaks indicative of a successful phage amplification event which require live bacteria. Thus, these strains would be accurately identified as *S. aureus* and would be deemed susceptible to cefoxitin. The MRSA strains BAA-1720 and BAA-1750 show phage amplification in both the no-antibiotic and 4 μg/mL preparations, successfully identifying each strain as *S. aureus* and determining its susceptibility to cefoxitin.

While detection of bacteria using PAD detected by MALDI-MS has been shown previously, to our knowledge, this is the first study showing the ability to ascertain antibiotic susceptibility by PAD as detected by MALDI-MS. Moreover, this is the first study using tryptic peptides of amplified phage proteins to detect phage amplification by MALDI-MS. In our experience, high-mass (>20 kDa), intact proteins derived from bacteriophage cannot always be detected by MALDI-MS, regardless of phage concentration. For example, while the capsid protein for *S. aureus* bacteriophage 53 is readily detected by MALDI-MS with basic sample preparation techniques [4], we have been unable to detect the intact major phage capsid protein for phage K by MALDI-TOF-MS regardless of sample preparation techniques, which include a reduction of disulfide bonds and sonication. However, with all bacteriophages in our laboratory, we have been able to detect tryptic peptides of phage proteins following trypsin digestion regardless of the bacteriophage in question.

We believe that demonstration of PAD on MALDI-MS instrumentation to detect bacteria and determine antibiotic susceptibility is a significant and novel step forward in integrating PAD for everyday clinical use. The use of trypsin digestion prior to MALDI-MS analysis means that many hard-to-detect (by MALDI-MS) bacteriophages using whole, intact virus analysis can likely be used in a PAD scheme where MALDI-MS is used as a detector. In our experience, we have been able to detect proteins from all bacteriophages at significant quantities using trypsin digestion prior to MS analysis, whereas MALDI-MS of whole, intact viruses often fails to detect any virus-derived proteins. We anticipate the continued acceptance of commercial MALDI-MS instrumentation for bacterial diagnostics and believe that PAD reagents and methodologies can be integrated into instruments already in place in microbiology and clinical laboratories. While MALDI-MS instrumentation is already capable of identifying bacteria from pure culture, PAD by MALDI-MS adds the component of antibiotic susceptibility determination to the MALDI-MS platform. As we have already demonstrated, PAD can be utilized to detect susceptibility to multiple antibiotics [7]. PAD in clinical samples can be conducted in multiple-well formats, lending itself to the processing of multiple samples in parallel and subsequent spotting on MALDI plates prior to analysis. Because MALDI-MS functions as a universal detector, obviating the need to develop and validate antibody-based detection systems, PAD kits can be added directly to existing MALDI-MS-based detection systems as they are developed and validated.

Several advantages combining PAD with MALDI-MS to identify bacteria and determine antibiotic resistance are apparent. First, while MALDI-MS currently relies upon the isolation of bacterial colonies prior to analysis, PAD can identify bacteria in mixtures [5], thereby significantly shortening the time to detection (5 h) because extended incubation times to visualize and obtain colonies can be avoided. Second, multiple antibiotics can be added to PAD workflows to obtain antibiotic susceptibility information against a panel of antibiotics. As phage replication is dependent upon a viable bacterial host, any agent that arrests growth or kills bacteria can be tested using PAD. Third, if a phage cocktail is utilized that contains typing phages, MALDI-MS can identify by mass which respective phage is amplified, thereby providing additional epidemiological data.

The single major barrier to implementation of a phage-based assay, in our opinion, is obtaining a bacteriophage or formulating a phage cocktail that has the requisite sensitivity and specificity. Phage detection systems do exist that incorporate a single bacteriophage; for example, the gamma phage lysis test to detect *Bacillus anthracis* is widely used in cases of anthrax outbreaks [15]. Contrariwise, the KeyPath MRSA/MSSA blood culture test incorporates a phage cocktail to obtain suitable sensitivity and specificity [16, 17]. Phage K, utilized in the present study, has a broad host range for many strains of *S. aureus* yet has been shown to also infect a small number of *Staphylococcus epidermidis* strains [18]. Thus, if phage K was to be incorporated into an assay, it would have to be augmented with other *S. aureus* bacteriophages, and the cross-reactivity to *S. epidermidis* would have to be mitigated, for example by the addition of the iron chelator deferoxamine to inhibit the growth of *S. epidermidis* strains [19].

While this study lays the methodological foundation for a PAD assay using MALDI-TOF-MS to identify *S. aureus* and determine antibiotic susceptibility, a robust validation must occur before moving to clinical implementation. Many more strains of bacteria including MSSA, MRSA, and common contaminating bacteria such as *S. epidermidis* must be tested to ensure broad-enough coverage while minimizing false positives. Importantly, *S. aureus* strains with MICs near the CLSI cefoxitin susceptibility (<4 μg/mL) and resistance (<8 μg/mL) values should be represented. Because of the ease of modifying this method, moving to a cutoff of 8 μg/mL cefoxitin may be warranted as more *S. aureus* strains are tested to ensure strains of intermediate resistance are properly assigned accurate susceptibility status. For the purposes of a PAD assay, the proper concentration of cefoxitin used must be determined empirically. For example, the FDA-approved KeyPath phage amplification detection methodology used 2 μg/mL cefoxitin as the cutoff point in its assay [16, 17].

## Conclusion

PAD followed by trypsin digestion and MALDI-MS analysis can identify *S. aureus* in samples and determine antibiotic susceptibility. PAD methodologies can be implemented on MALDI-MS instrumentation already available in many healthcare settings. The next step in incorporating PAD with MALDI-MS is the development of a phage or phage cocktail that has a broad-enough host range to generate the necessary sensitivity and specificity to provide actionable information. MALDI-MS is an ideal platform for PAD as it functions as a universal detector, expediting the research and development of new phage/host systems as it makes antibody-based detection platforms unnecessary. Further, phage/host systems for numerous bacteria can be deployed on MALDI-MS systems already in place in microbiology laboratories.

## Electronic supplementary material

Below is the link to the electronic supplementary material.

ESM 1 (PDF 135 kb)
