# Supplementary material for: Detection of methicillin-resistant Staphylococcus aureus using phage amplification combined with matrix-assisted laser desorption/ionization mass spectrometry
Source: Anal Bioanal Chem. 2016 Nov 19;409(5):1379–86. doi: 10.1007/s00216-016-0070-3 (PMC5258805; doi:10.1007/s00216-016-0070-3)
Supplement: Supplementary file 1 — (PDF 135 kb) [file 216_2016_70_MOESM1_ESM.pdf]

Analytical and Bioanalytical Chemistry

Electronic Supplementary Material

Detection of methicillin-resistant *Staphylococcus aureus* using phage amplification combined with matrix-assisted laser desorption/ionization mass spectrometry

Jon C. Rees, John R. Barr

Table S1 List of all peptide sequences found for a tryptic digest of Phage K by LC-MS/MS followed by database searching. Peptides used for MALDI-MS identification of Phage K are highlighted in yellow

Accession gi|48696433	Description - Putative capsid protein
Sequence	Actual Mass
(K)SFQTGYGITPDTQIDAGALR(R)	2,110.03
(K)EIGVAPVSDPNIR(Q)	1,365.73
(K)NMSIASGLVNNIADPSQILTEDAIAVVAK(T)	2,953.55
(K)TIEWASFYGDASLTSEVEGEGLEFDGLAK(L)	3,120.45
(K)HLNEAAVR(I)	908.4836
(K)GFGTATDAYMPIGVHADFVNSILGR(Q)	2,608.29
(K)LHGSTVMENELILDESLQPLPNAPQPAK(V)	3,040.56
(K)GAFENEEDR(A)	1,065.44
(K)LSINVNAMYQQQPQFVSIYR(Q)	2,398.22
(K)DAQEDGTIVFVDK(N)	1,435.68
Accession gi|48696453	ORF65
Sequence	Actual Mass
(K)VLFNPDRPLQQAELNEMQSIDQYYLK(N)	3,151.58
(R)IVTPDEDASLLDQTSGVPSYFSK(G)	2,468.21
(K)DYEVTTQGEGLSK(K)	1,425.66
(R)TLEEMIEFIR(I)	1,279.65
(K)GSEDGTIMTDAK(G)	1,223.54
(K)NANSTSATTYTAQGR(K)	1,541.71
(R)VTVNLVDPLAQSFQYDENR(T)	2,207.09
(K)WEPIGNYQDLDVLGLAR(Q)	1,957.99

Accession gi|48696481	ORF95
Sequence	Actual Mass
(K)NVNDVLQQESVTVADK(Y)	1,757.88
(K)DSLEHAHEDVIADFPASPAR(K)	2,176.02
(K)GVDSGEGQGDTSEPSAPK(N)	1,716.74
Accession gi|48696454	ORF66
Sequence	Actual Mass
(K)GAPSGEATAGFVR(L)	1,218.60
(K)ELTESTTQYATLVNPPK(E)	1,890.96
(R)EITASEITTEWK(K)	1,406.69
(K)GNGAVVYYALPK(L)	1,250.66
Accession gi|48696456	ORF68
Sequence	Actual Mass
(M)ALNFTTITENNVIR(D)	1,604.86
(R)DDGSASFPLLVYTSDSK(T)	1,800.85
(K)TFQQAIIDHIDR(T)	1,455.75
(K)ELWTGAQSFLSTGTTK(N)	1,725.86
(K)GSTWTLVDSAVQNSK(T)	1,591.79
Accession gi|48696432	ORF43
Sequence	Actual Mass
(K)EAQEQGEKVESEEVAEGNEDEEVEK(S)	2,819.22
(K)VESEEVAEGNEDEEVEK(S)	1,919.82

Accession gi|48696452	ORF64
Sequence	Actual Mass
(K)WVYLESSIVGDELPLGTYR(Q)	2,196.12
(K)FNLVPSEVESTGTLLFFDNK(Q)	2,256.14

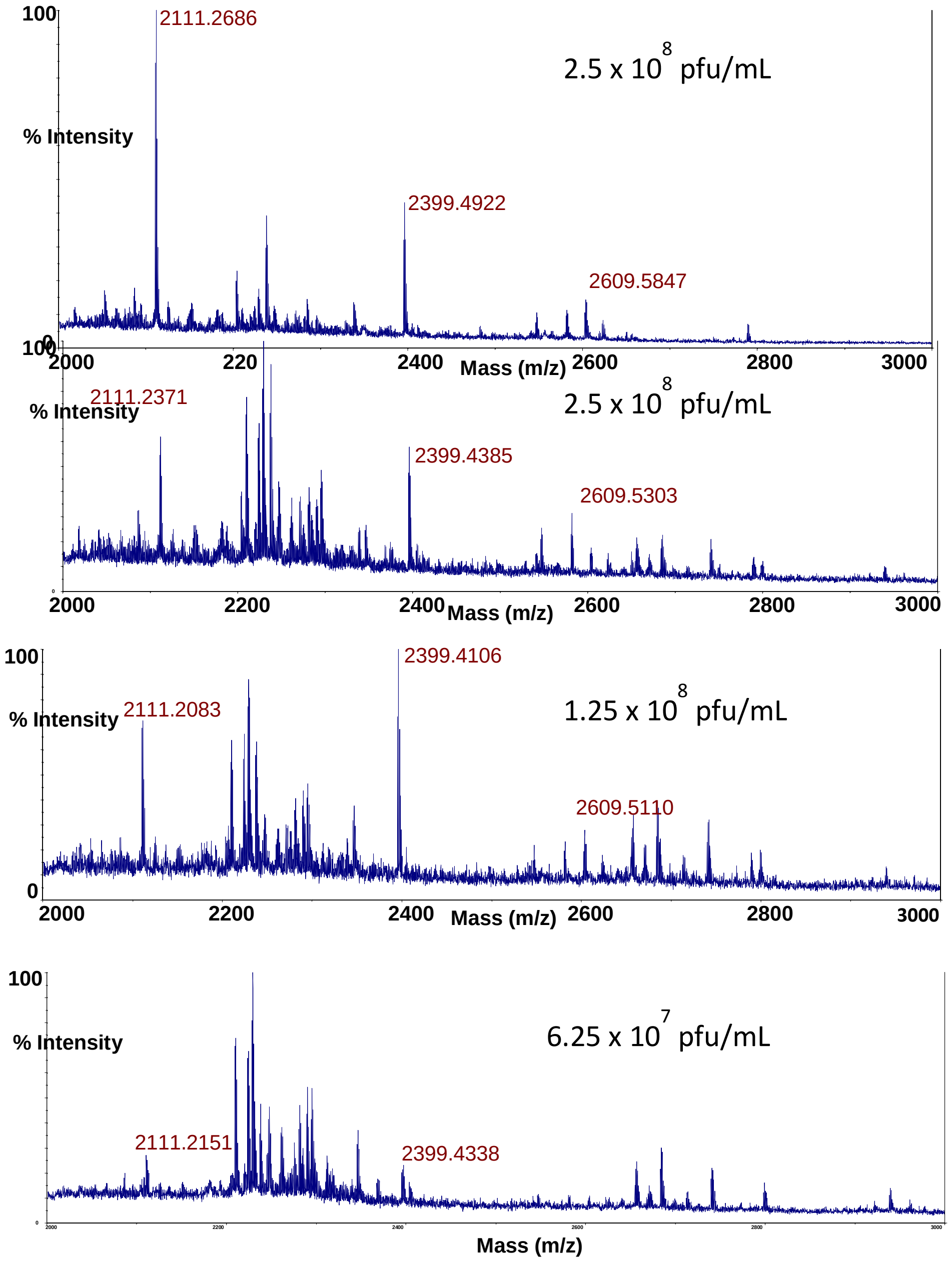

Fig. S1 Dilution series of bacteriophage K as detected by MALDI-MS

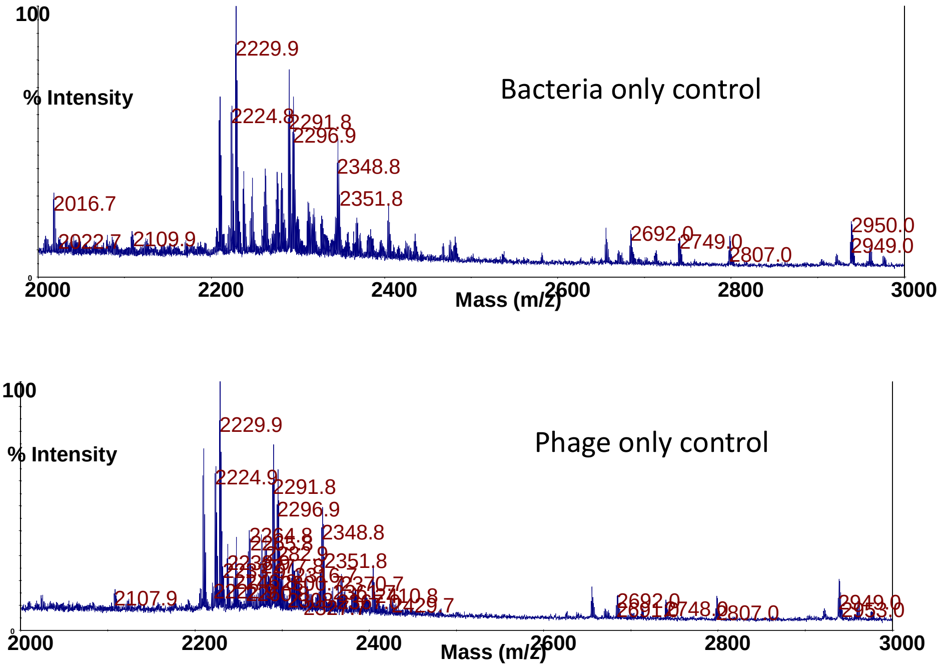

Fig. S2 Control spectra from samples containing bacteria only and phage only processed after a 5 hour incubation. Peaks associated with phage amplification cannot be found in either spectrum
